# Perinatal Deaths in Suriname: A Nationwide Cohort Study on Causes of Death and Lessons Learned from Facility-Based Audits

**DOI:** 10.1007/s10995-026-04280-1

**Published:** 2026-05-14

**Authors:** Zita D. Prüst, Kim J. C. Verschueren, Safir Liesdek, Fernando Rigters, Gieta Bhika-Kori, Kitty W. M. Bloemenkamp, Thomas van den Akker, Lachmi R. Kodan

**Affiliations:** 1 Department of Obstetrics, Division Women and Baby, Birth Centre Wilhelmina’s Children Hospital, University Medical Centre Utrecht, Utrecht University, Postbus 85090, 3508 AB Utrecht, The Netherlands; 2 Department of Obstetrics and Gynecology, Academic Hospital Paramaribo (AZP), Flustraat, Paramaribo, Suriname; 3 Department of Obstetrics and Gynecology, Leiden University Medical Center, Postbus 9600, 2300 RC Leiden, The Netherlands; 4 Department of Neonatology, Academic Hospital Paramaribo (AZP), Flustraat, Paramaribo, Suriname; 5 Department of Obstetrics and Gynecology, ‘s Lands Hospitaal Paramaribo Suriname, Tourtonnelaan 3, Paramaribo, Suriname; 6 Anton de Kom University of Suriname, Leysweg, Paramaribo, Suriname

**Keywords:** Perinatal mortality, Cause of death, Audit, MPDSR, Suriname, Middle-income country

## Abstract

**Objectives:**

The WHO recommends implementing the Maternal and Perinatal Death Surveillance and Response (MPDSR) cycle to evaluate and reduce perinatal mortality. We aimed to (1) assess Suriname’s national perinatal mortality rate (PMR), (2) classify perinatal death causes, (3) investigate contributing modifiable factors.

**Methods:**

A nationwide, prospective cohort study of perinatal deaths in Suriname (June - December 2022). We conducted surveillance, classified causes of death and conducted audits, as part of the MPDSR cycle. We audited a random selection of cases and used quantitative analysis to report modifiable factors.

**Results:**

We identified 83 (53%) stillbirths and 72 (47%) neonatal deaths. The PMR was 28.2 per 1000 total births. Stillbirths occurred antenatally in 80% (*n* = 66/83), of which 49% (*n* = 32/66) of unknown cause. Leading causes of antepartum stillbirth were placental insufficiency and placental abruption (combined 37.8%, *n* = 25), often linked to hypertensive disorders of pregnancy (HDP) (*n* = 19). In 23 neonatal death cases, missing files hindered cause classification. Congenital anomalies (29%, *n* = 14/49) and infections (22%, *n* = 11/49) were primary causes of neonatal deaths. We reviewed 63 cases and identified 150 modifiable factors, half of which related to quality-of-care, including suboptimal antenatal care and substandard neonatal life support. Delays in seeking care and shortages of supplies and staff also contributed significantly.

**Conclusions:**

To decrease perinatal mortality, we suggest enhancing antenatal and neonatal care, by improved ultrasound, better triage, mitigating the impact of HDP, and upgrading neonatal respiratory support. Furthermore, policymakers should prioritize improving access to care, availability of trained staff and sustainable implementation of the MPDSR cycle.

**Supplementary Information:**

The online version contains supplementary material available at 10.1007/s10995-026-04280-1.

## Significance

What is Already Known About this Subject?

What this Study Adds?

The WHO recommends implementation of perinatal mortality audits, as part of the Maternal and Perinatal Death Surveillance and Response cycle, to understand *how* and *why* perinatal deaths occur and establish local recommendations. However, in many countries with high perinatal mortality, mortality audits are not conducted.

This article serves as an example for implementing audits in similar settings. We showed that audits are feasible in middle-income country Suriname and provide tailor-made recommendations to improve quality-of-care. To reduce perinatal mortality in Suriname, policymakers should prioritize improving access to care, enhancing antenatal and neonatal care quality, and increasing trained staff availability.

**Supplementary Information:**

The online version contains supplementary material available at 10.1007/s10995-026-04280-1.

## Introduction

Annually, 2.6 million infants are stillborn, and another 2.9 million die within the first month of life (World Health Organization, 2014; WHO, 2024a, 2024b). The vast majority (98%) of these deaths occur in low- and middle-income countries (LMIC), where access to quality skilled care before, during, and after birth is often limited (WHO, 2014).

The World Health Organization’s (WHO) *Every Newborn Action Plan (ENAP)* aims to reduce perinatal mortality globally and achieve a target set for 2035 of no more than 10 stillbirths per 1000 total births and less than 10 neonatal deaths per 1000 live births in every country (WHO, 2014). To realize this goal, the WHO advocates for implementation of the Maternal and Perinatal Death Surveillance and Response (MPDSR) cycle (WHO, 2021). The MPDSR is a continuous loop of identifying cases, collecting data, conducting mortality audits, pinpointing and implementing quality-of-care improvements and assessing the effectiveness of implemented measures. By implementing the MPDSR, valuable insights into the causes and modifiable factors contributing to perinatal deaths can be obtained. This information can inform decision-makers to enact tailored interventions and foster positive quality-of-care changes that can contribute to prevention. However, in most countries where mortality is high, the MPDSR is not in place (WHO, 2021; United Nations International Children’s Emergency Fund, 2019).

Suriname is an example of a country where the MPDSR cycle is not implemented and consequently, the incidence, causes and contributing factors to perinatal deaths are not completely understood. With 14.8 stillbirths per 1000 total births (2017), Suriname has one of the highest stillbirth ratios (SBR) in the Latin American/Caribbean region (Prüst et al., 2020; Verschueren et al., 2020). Previous investigation into stillbirths in Suriname showed that the majority occur during the antepartum period due to hypoxia and maternal hypertensive disorders (Prüst et al., 2020). However, the incidence and causes of neonatal mortality remain unknown, and the contributing modifiable factors are not yet studied. The next step for a better understanding of perinatal mortality in Suriname is to introduce the MPDSR cycle.

This nationwide prospective perinatal death study aimed to (1) assess the national perinatal mortality rate (PMR), (2) classify the causes of perinatal deaths and (3) investigate modifiable factors that contribute to perinatal deaths in Suriname.

## Methods

### Study Design

This is a nationwide, prospective cohort study of perinatal deaths between June 2022 and December 2022, representing the introduction of the MPDSR cycle in Suriname. We included every perinatal death in the country, collecting data from the hospitals, primary healthcare centers and vital statistics. We retrieved data from the medical files, and classified the causes of death according to the WHO International Classification of Diseases – Perinatal Mortality (ICD-PM) (WHO, 2016). Additionally, we conducted facility-based audit and summarized lessons learned.

### Setting

Suriname, an upper-middle-income country on the northeast coast of South America, has a population of 618,040 (2022) (World Bank, 2024). About 90% of the population lives in the capital or along the coastline, where healthcare facilities are easier accessible. The population living in rural areas of the interior rainforest face long travel times to reach healthcare facilities due to poor infrastructure (World Bank, 2024; Ministry of Health Suriname, 2021; Kodan et al., 2017). The country has five hospitals: four in the capital Paramaribo and one on the west coast in Nickerie. All five hospitals provide obstetric and neonatal care, four with general pediatric wards and one with a Neonatal Intensive Care Unit (NICU). Additionally, there are about 100 primary healthcare centers in rural areas that offer antenatal, labor and postpartum care, in general only to low-risk women (Ministry of Health Suriname, 2021; Kodan et al., 2017). Annually, about 10,000 live births occur, with 90% in hospitals and 3% in primary health centers (Ministry of Health Suriname, 2021). Women with pregnancy or birth complications are referred to a hospital. Vital statistics from the Central Bureau of Civil Affairs (CBB) include neonatal deaths but not stillbirths. In 2017, the Stillbirth Rate (SBR) was 14.4 per 1000 births, and the Neonatal Mortality Rate (NMR) was 12.0 per 1000 live births (Prüst et al., 2020; Ministry of Health Suriname, 2021). The viability limit in Suriname is 27 weeks of gestation or 750 g of birthweight, though survival rates by gestational age are unknown. Perinatal deaths are not reviewed by a national audit committee. More details on the health system are available in previous studies (Prüst et al., 2020; Verschueren et al., 2020; Kodan et al., 2017).

### Variables and Definitions

Perinatal death is the sum of stillbirths and neonatal deaths (WHO, 2014). Stillbirth was defined as an infant born with no signs of life at a minimum of 27 weeks’ gestation or a minimum of 750 g birthweight. Neonatal death was defined as an infant born alive but that died within the first 28 days of life, with the first 24 h of life as ‘day 1’. Early neonatal death was defined as a neonatal death within the first 7 days of life (WHO, 2014, 2016).

To facilitate international comparisons, we defined the SBR according to the WHO definition: the number of late stillbirths (*≥* 28 weeks of gestation and/or 1000 g birthweight) per 1000 total births (WHO, 2024b). Over the study period, 4937 total births (stillbirths and livebirths) with a gestation of *≥* 28 weeks were registered by the vital statistics (Algemeen Bureau voor Statistiek, 2021). The NMR was defined as the number of neonatal deaths (up to 28 days of life) per 1000 live births (WHO, 2024a). Over the study period, 4932 live births (regardless of the gestational age) were registered by the vital statistics (Algemeen Bureau voor Statistiek, 2021). The perinatal mortality rate (PMR) was defined as the sum of late stillbirths and early neonatal deaths (within the first 7 days) per 1000 total births *≥* 28 weeks of gestation.

The onset of labor was defined iatrogenic if there was a record of induction of labor or cesarean section before the start of spontaneous labor. Birthweight percentiles were defined based on the Dutch Perined (Hoftiezer) birthweight curves (Perined, 2023). Fetal growth restriction (FGR) was defined as a birthweight under the 10th percentile. Preterm birth was defined as birth before 37 weeks of gestation (WHO, 2014). Ethnicity was self-reported by the woman.

### Data Collection

#### Identification

First, we identified perinatal deaths by a weekly visit to the maternity ward and pediatric ward (including the NICU) in all five hospitals. We screened the parturition books and patient administration books and questioned the midwives and doctors in charge on their knowledge of new cases of perinatal deaths. If it was unclear whether an infant was born dead or alive, we examined the medical file. Second, a monthly check of the parturition books in the primary healthcare organizations was done. Finally, we cross-checked our inclusions with the neonatal deaths identified by The Central Bureau of Civil Affairs during the national vital statistics and included any missing cases. One author (ZP) carried out the identification process. Identification numbers were crosschecked to prevent double inclusion, such as when a woman gives birth at one healthcare facility but the infant dies at another.

#### Data Collection

We reviewed the medical files of every perinatal death and conducted verbal autopsy with the birth attended or healthcare provider during antenatal care and/or neonatal care in case of a missing file. Medical records were obtained from the hospitals and supplemented with information from primary care facilities when available. We gathered all available information (i.e., laboratory results, pathology reports, parturition books, verbal autopsy) to extract variables according to the WHO stillbirths and neonatal death case review form (WHO, 2021). An elaborated case summary was made for every perinatal death.

#### Causes of Death Classification

We classified every perinatal death (by at least two independent clinicians) according to the WHO ICD-PM, by assigning three conditions: (1) the timing of death, (2) the cause of death, and (3) the contributing maternal condition(s) (supplementary file 1) (WHO, 2016). We distinguished between antepartum and intrapartum stillbirth by using the presence of fetal heart rate (FHR) during admission, the presence of painful uterine contractions, and information on cervical dilation. The FHR was confirmed using CTG, and if no fetal heart rate was detected, this was verified by ultrasound. Maceration was not used as an indicator of the timing of death (Gold et al., 2014). If the timing of the stillbirth was unclear, we classified the death as a *stillbirth of unknown timing*. Common causes of death were classified uniformly throughout the study. For instance, we classified antepartum stillbirth of a growth-restricted fetus among women with severe pre-eclampsia as A3 ‘antepartum hypoxia’ and M4 ‘maternal medical and surgical conditions’. We classified antepartum stillbirth after placental abruption among women with severe pre-eclampsia as A4 ‘Other specified antepartum disorder’ combined with M1 ‘Complications of placenta and membranes’ and M4 ‘maternal medical and surgical conditions’ (supplementary file 2). The level of certainty was categorized as ‘uncertain’, ‘possible’, ‘probable’, or ‘certain’. Disagreements were resolved through consultation with the senior authors (TvdA and/or KB).

#### Perinatal Mortality Audit

Due to the high caseload and duration of the audit sessions, we randomly selected 41% (63/155) of the perinatal death cases for review. A local expert committee, including obstetricians, midwives, pediatric nurses, and pediatricians from various hospitals, reviewed these cases. Each audit session had one moderator. To ensure a secure environment, three rules were followed: (1) the meeting is confidential, (2) all caregivers were recognized as experts in their own field, and (3) participants were encouraged to pose inquisitive, non-judgmental questions. Audits were conducted within a multidisciplinary team to encourage collective reflection rather than focus on individual accountability. The emphasis was placed on identifying system-level factors rather than individual ones. Hospital leadership was involved from the outset to support the audit process and foster a culture of open discussion.

Cases were presented chronologically and assessed for positive points and improvable care (modifiable factors), which are defined as factors that contributed to an adverse event and could be altered in future situations. We used the six “what” questions with the aim to conduct root cause analysis (supplementary file 3) (Van Diem et al., 2012). Root cause analysis is founded on the principle that the presence of a modifiable factor is rarely solely attributable to one individual but is typically the outcome of a variety of underlying causes. Modifiable factors were categorized into four groups: (1) patient/family-related, (2) team/communication-related, (3) quality of care-related, and (4) system/management-related. Multiple factors could be assigned to a single case. Local guidelines or, if unavailable, standard practices defined by local clinicians were used as benchmarks. Modifiable factors were reported, but recommendations yielded from the audits were not yet implemented during this study period.

### Analysis

Data were manually entered into Microsoft Excel and IBM SPSS version 29.0 for analysis. We applied descriptive statistics using numbers, percentages, means, and standard deviations and summarized them in tables.

### Ethical Approval

This research adhered to the principles outlined in the Declaration of Helsinki and received approval from the ethical review board of the Surinamese Committee on Research Involving Human Subjects (CMWO 0322).

## Results

### Identification

**Fig. 1:**
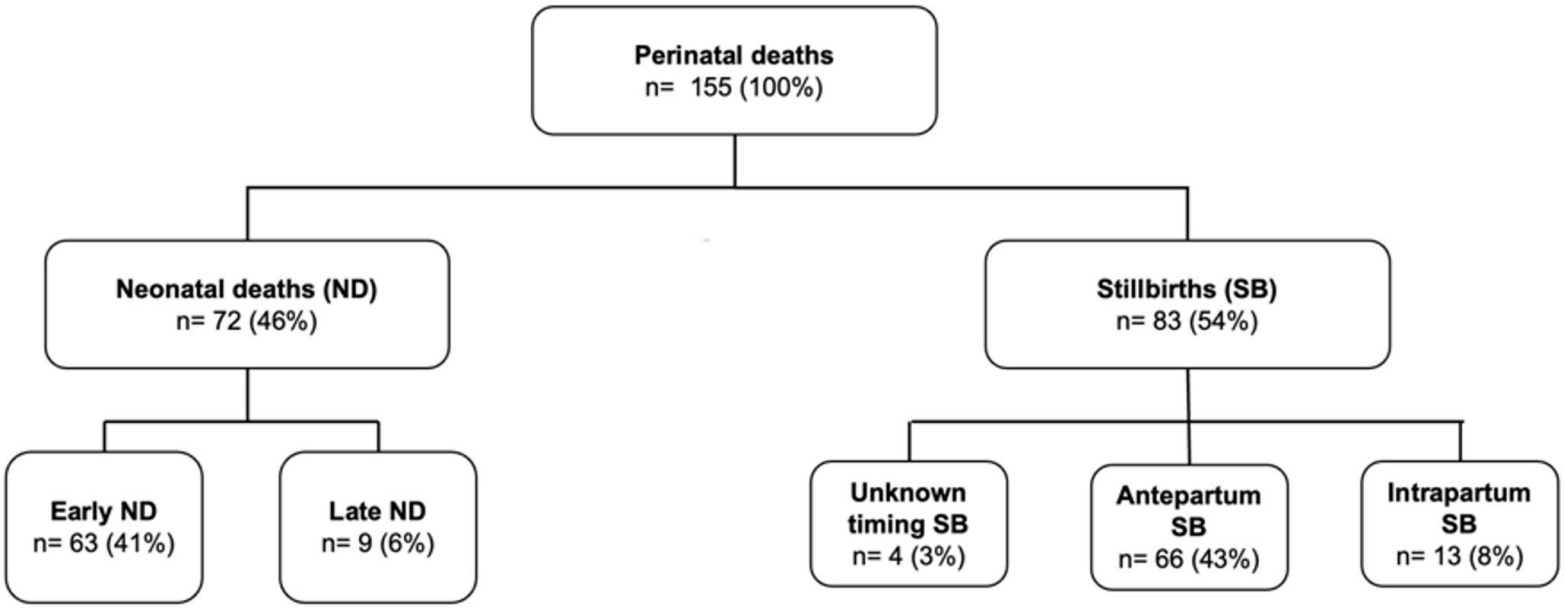
Flowchart of perinatal deaths in Suriname, June 2022–December 2022

We identified 155 perinatal deaths: 83 (53.5%) stillbirths and 72 (46.5%) neonatal deaths (Fig. 1). There were 76 stillbirths *>* 28 weeks of gestation, resulting in a SBR was 15.8 per 1000 total births (76/4937 total births *>* 28 weeks of gestation). The NMR was 14.6 per 1000 live births (72/4932 live births). There were 63 early neonatal deaths (within the first seven days of life). The PMR was 28.2 per 1000 total births (139/4937 total births *>* 28 weeks of gestation). Perinatal deaths occurred in a hospital in 97.4% (*n* = 151) and within a primary healthcare facility in 2.4% (*n* = 4). No perinatal deaths occurred during transport after referral to another facility. Most of the stillbirths (79.5%, *n* = 66/83) were during the antenatal period.

We identified 132 perinatal death cases through the weekly screening in the hospitals and primary healthcare facilities and 23 neonatal death cases through the national vital statistics. For these 23 cases, there was no medical file, or it could not be identified, and so further analysis and cause of death classification was not possible.

Table 1 shows the baseline characteristics of the 132 cases that were included for analysis (Table 1). In 16.2% (*n* = 21) of these cases, women experienced a perinatal death before. Women received an antenatal care check-up within the first trimester of pregnancy in 26.7% (*n* = 32) and an ultrasound within the first trimester of pregnancy in 20.3% (*n* = 25). In 25.0% of all cases (*n* = 33) the birthweight percentile was lower than 10, of which less than one-third (*n* = 9) were diagnosed antenatally. In 24.2% (*n* = 32) the birthweight percentile could not be determined due to an unknown gestational age at the moment of death.

**Table 1:** Baseline characteristics

Total	Total perinatal deaths	Stillbirths	Neonatal deaths
	*n* = 132	*n* = 83	*n* = 49
**Maternal age (years)**			
< 20	17 (13.0)	7 (8.4)	10 (20.8)
20–35	92 (70.2)	61 (73.5)	31 (64.6)
*≥* 36	22 (16.8)	15 (18.1)	7 (14.6)
*Missing*	*1*		*1*
**Women’s educational level**			
None	2 (3.3)	0	2 (9.5)
Primary/Secondary education	50 (83.3)	36 (92.3)	14 (66.7)
Higher education	8 (13.3)	3 (7.7)	5 (23.8)
*Missing*	*72*	*44*	*28*
**Women’s profession**			
Formally employed	29 (39.7)	17 (37.0)	15 (55.6)
Unemployed	44 (60.3)	29 (63.0)	12 (44.4)
*missing*	*59*	*37*	*22*
**Insurance**			
None	16 (12.4)	14 (17.3)	2 (4.2)
State Health Insurance	101 (76.5)	59 (72.8)	42 (87.5)
Private Insurance	12 (9.1)	8 (9.9)	4 (8.3)
*missing*	*3*	*2*	*1*
**Antenatal care visits**			
None	20 (16.7)	15 (19.7)	5 (11.4)
< 4	36 (30.0)	22 (28.9)	14 (31.8)
*≥* 4	64 (53.3)	39 (51.3)	25 (56.8)
*missing*	*12*	*7*	*5*
**Antenatal ultrasound**			
None	21 (17.1)	17 (21.8)	4 (8.9)
*≥* 1 before 24 weeks of gestation	76 (61.8)	43 (55.1)	33 (73.3)
*≥* 1 after 24 weeks of gestation	26 (19.7)	18 (23.1)	8 (17.8)
*missing*	*9*	*5*	*4*
**Parity**			
0	36 (27.5)	18 (22.0)	18 (36.7)
1–3	64 (48.9)	39 (47.6)	25 (51.0)
*≥* 4	31 (23.7)	25 (30.5)	6 (12.2)
*missing*	*1*	*1*	*-*
**History of perinatal death**			
Yes	21 (16.2)	14 (17.1)	7 (14.3)
*Missing*	*2*	*1*	*1*
**Type of pregnancy**			
Twins	8 (6.1)	5 (6.0)	3 (6.1)
**Gestational age (weeks)**			
< 28 weeks	14 (10.6)	5 (6.0)	9 (18.4)
28–31	53 (40.2)	31 (37.3)	22 (44.9)
32–36	28 (21.2)	22 (26.5)	6 (12.2)
*≥* 37	37 (28.0)	25 (30.1)	12 (24.5)
**Birthweight (percentile)**			
Unable to determine	32 (24.2)	27 (32.5)	5 (10.2)
< p10	33 (25.0)	19 (22.9)	14 (28.6)
p10 – p90	57 (43.2)	31 (37.3)	26 (53.1)
> p90	10 (7.6)	6 (7.2)	4 (8.2)
**Mode of birth**			
Spontaneous, vaginal	112 (86.2)	75 (91.5)	37 (77.1)
Ventouse	1 (0.8)	0	1 (2.1)
Elective caesarean	8 (6.2)	2 (2.4)	6 (12.5)
Emergency caesarean	9 (6.9)	5 (6.1)	4 (8.3)
*missing*	*2*	*1*	*1*
**Level of certainty for cause of death**			
Uncertain	22 (16.7)	19 (22.9)	3 (6.1)
Possible	12 (9.1)	12 (14.5)	0 (0)
Probable	49 (37.1)	21 (25.9)	28 (57.1)
Certain	37 (28.0)	19 (22.9)	18 (36.7)
**Maternal death**			
Yes	1	1	0

Preterm birth occurred in 72.0% (*n* = 95) of the included cases. Among the neonatal deaths (*n* = 49) preterm birth (*n* = 37/49, 75.5%) was iatrogenic in 32.4% (*n* = 12) and spontaneous in 67.6% (*n* = 25).

### Causes of Perinatal Death

Table 2 shows the causes of perinatal death (see also supplementary file 2 for elaborated ICD-PM classification) (Table 2). Nearly half (48.5%, *n* = 32) of the antepartum stillbirths were of unknown cause. Placental insufficiency and placental abruption were the most common causes of antepartum stillbirth (together 37.8%, *n* = 25), among which most women had a hypertensive disorder of pregnancy (HDP) (*n* = 19). Placental abruption was the cause of intrapartum stillbirth in 46.2% (*n* = 6). Of all included cases, 31.1% (*n* = 41/132) of the women had HDP. The cause of death for stillbirths was ‘certain’ in 22.9% (*n* = 19) and ‘probable’ in 25.9% (*n* = 21) (Table 1).

**Table 2:** Causes of perinatal mortality in Suriname

Cause of death	Frequency	Per cent
**Antepartum stillbirths (n = 66)**		
Unknown cause	32	48.5
Placental insufficiency (with and without HDP^a^)	13	19.7
Placental abruption (with and without HDP)	12	18.2
Congenital anomalies	4	6.1
Complications of cord or membranes^b^	2	3.0
Gestational diabetes	2	3.0
Other severe maternal disease^c^	1	1.5
**Intrapartum stillbirths (n = 13)**		
Placental abruption	6	46.2
Intrapartum asphyxia	2	15.4
Uterine rupture	2	15.4
Congenital anomalies	1	7.7
Intra-uterine infection	1	7.7
Unknown cause	1	7.7
**Stillbirth of unknown timing (n = 4)**		
Unknown cause	4	100
**Neonatal deaths (n = 49)**		
Congenital anomalies^d^	14	28.6
Infections	11	22.4
Prematurity	7	14.3
Respiratory distress syndrome	7	14.3
Intrapartum asphyxia	5	10.2
Aspiration	2	4.1
NEC	2	4.1
Meconium aspiration syndrome	1	2.0

^a^Hypertensive disorder of pregnancy

^b^Prolapsed cord (*n* = 1), PPROM and premature contractions (*n* = 1)

^c^SLE (*n* = 1)

^d^hernia diaphragmatic (*n* = 4), gastroschisis (*n* = 1), anus and esophagus atresia (*n* = 2), cardiac (*n* = 2), hydrocephalus (*n* = 1), multiple cong. (*n* = 4)

Neonatal deaths were most frequently due to congenital anomalies (28.6%, *n* = 14). Approximately one-third (5/14) of these congenital anomalies were diagnosed antenatally. The second most common cause of neonatal deaths was infections (22.4%, *n* = 11) among which one case with prolonged premature rupture of membranes and clinical signs of chorioamnionitis. The cause of death of neonatal deaths was ‘certain’ in 36.7% (*n* = 18) and ‘probable’ in 57.1% (*n* = 28) (Table 1).

### Perinatal Audit

We reviewed 63 cases, of which 38 stillbirths (60.3%) and 25 (39.7%) neonatal deaths. We identified one or more modifiable factors in 62 cases (99.2%) and assigned a total of 150 modifiable factors. In 23.8% of cases (*n* = 15/63) there was 1 modifiable factor, in 23.8% (*n* = 15/63) there were 2 modifiable factors and in 50.8% (*n* = 32/63) there were 3 or more modifiable factors.

The assigned modifiable factors are summarized in Table 3 (Table 3). Half of these were related to quality of care (50.0%, *n* = 75). The audit committee found that improvement during antenatal care can specifically be made in the detection of FGR (15 missed cases). Out of the 15 cases with a missed diagnosis of FGR, 8 women had an ultrasound within 2 weeks before birth. Other improvements during antenatal care could be made in more frequent check-ups and ultrasounds among high-risk women and fetuses, better monitoring of hypertension, and the use of acetylsalicylic acid and calcium for women with high risk of HDP.

**Table 3:** Modifiable factors assigned by 63 out of 155 perinatal deaths, divided into groups and subgroups

Modifiable factor	*n*	(%)	Subgroup	*n*
Patient/family related	28	18.7		
			First delay (seeking care)	27
			Adherence to therapy	1
Team/communication related	25	16.7		
			Communication between primary and secondary healthcare facility	9
			Documentation	6
			Communication between different departments within same facility	5
			Communication within the same team	1
Quality of care	75	50.0		
			Diagnosis of Foetal Growth Restriction (FGR)	15
			Quality and content of antenatal care check-ups	15
			Incomplete treatment by clinician	9
			Newborn life support	8
			Other diagnoses or additional diagnostics	7
			Foetal monitoring during birth	6
			Timely clinical examination of patient during hospital admission	4
			Timely decision for induction/cesarean section	4
			Triage between high-risk and low-risk women	4
			Diagnosis of foetal congenital anomalies during the prenatal phase	2
			Recognition of neonatal sepsis	2
			Recognition of start of delivery	2
			Management of hypertensive disorders of pregnancy during admission	1
System/management related	22	14.7		
			Possibility for neonatal admission due to shortage of available staffed NICU beds	9
			Possibility to provide newborn respiratory support	6
			Equipment for foetal monitoring	3
			Local guidelines	2
			Transport from primary to secondary healthcare	2
Total	150	100		150

A delay in seeking care was found in 42.9% (*n* = 27) of the reviewed cases. Poor communication occurred in 23.8% (*n* = 15) and was most frequently between primary and secondary healthcare facilities (*n* = 9).

Key modifiable factors during the early neonatal phase were suboptimal quality of newborn life support (*n* = 8), the inability to provide neonatal respiratory support (*n* = 6), and the impossibility of neonatal hospital admission due to shortage of available staffed NICU beds (*n* = 9). The latter two due to a shortage of staff and supplies.

## Discussion

The PMR in Suriname is 28 per 1000 total births in 2022 Of all perinatal deaths, 72% (*n* = 95/132) occurred preterm. Stillbirths predominantly occur antepartum, with the cause remaining unknown in nearly half of the cases. When a cause is identified, it is often linked to placental insufficiency and placental abruption among women with HDP. Neonatal deaths are most often within the first seven days of life and are primarily attributed to congenital anomalies and infections. Our audits revealed that half of the assigned modifiable factors were related to the quality of care such as inadequate quality of antenatal care (including timely diagnosis of FGR) and neonatal life support. Other important modifiable factors included a delay in seeking care, and the inability to provide neonatal respiratory support and neonatal hospital admission due to a shortage of staff and supplies.

Accurate perinatal mortality surveillance is imperative for targeting the areas with the greatest need and constitutes the initial step toward reducing perinatal deaths (WHO, 2021; Anwar et al., 2018). We identified 23 cases of perinatal deaths that occurred outside of healthcare facilities through a cross-check with national vital statistics. On the other hand, we found 10 perinatal death cases that were not registered by the national vital statistics. This finding is significant, as it suggests potential underreporting of perinatal mortality in previous national reports and emphasizes the importance of a multisource approach to enhance surveillance.^16^ To reduce underreporting, Suriname may benefit from adding community-based death notification and the enhancement of facility-based death surveillance (WHO, 2021; Anwar et al., 2018; Willcox et al., 2023).

Global goals aim for an SBR under 10 per 1000 total births and an NMR under 10 per 1000 live births in all countries by 2035 (WHO, 2014). Our study in Suriname found a higher SBR (15.8) and NMR (14.6), surpassing the WHO estimates (SBR 10.9 and NMR 10.6) (WHO, 2024a, 2024b). The SBR in our study is similar to the hospital SBR of 16 per 1000 total births reported in Suriname in 2016 and 2017 (Verschueren et al., 2020). The NMR has increased compared to the last figures reported by the National Bureau of Statistics (2018, NMR 12), possibly due to improved surveillance, the impact of the COVID-19 pandemic, and the economic recession (UNICEF, 2019). Implementing a national perinatal reduction strategy, that includes introduction of the MPDSR cycle, is crucial for Suriname to align with global goals and reduce perinatal deaths.

Reducing the incidence of unexplained stillbirths is challenging, particularly in low- and middle-income settings (UNICEF, 2019; Aminu et al., 2019). Placental pathology and fetal autopsy are key diagnostic tests (Page et al., 2017; Page & Silver, 2018). Suriname has a high rate of unexplained stillbirths (40% in this study, 41% in a previous study of Prüst et al. (2020)), which could be reduced with a national guideline for diagnostic work-up. Placenta pathology was conducted in only 18% (*n* = 15) of the stillbirth cases, and autopsy was not performed. As observed in many other countries, the feasibility of autopsy in Suriname is hindered by factors such as costs, aversion to invasiveness, religious and cultural beliefs (including burial practices), and organizational barriers (Lewis et al., 2018; O’Keefe et al., 2023). Increasing the frequency of placental pathology, especially for unknown stillbirths, is therefore a first step. Additionally, we emphasize the importance of modifiable factors such as communication (among healthcare professionals between and within facilities) and documentation (of clinical signs, vitals and diagnostics). Furthermore, early detection of FGR as a potential cause of death, holds promise in reducing the proportion of unexplained stillbirths in Suriname (Aminu et al., 2019).

Previous research in Suriname identified HDP as the main cause of stillbirth and perinatal morbidity (Prüst et al., 2020, 2021). This study confirms that when a cause is determined, it is predominantly associated with placental insufficiency and placental abruption in women with HDP. Notably, 31% (*n* = 41/132) of the women with a perinatal death had HDP. Our perinatal death audits revealed common modifiable factors including delays in seeking care, suboptimal quality of antenatal care, and insufficient recognition and referral of high-risk pregnancies. Improvement of HDP management during admission was assigned once. In light of these findings, we recommend strategic plans to mitigate the impact of HDP on adverse perinatal outcomes, such as improving the quality of antenatal care with a particular focus on adequate use of use of acetylsalicylic acid and calcium for prevention and early recognition and treatment of HDP during antenatal check-ups (LeFevre & U.S. Preventive Services Task Force, 2014; Hofmeyr et al., 2018). Furthermore, better triage of high-risk pregnancies, such as women with HDP or a history of perinatal death, is crucial.

Preterm birth is a major risk factor for neonatal mortality and morbidity (WHO, 2014; Simmons et al., 2010). In this study, over half (51.0%, *n* = 25/49) of the neonatal deaths were among premature infants after a spontaneous onset of birth. It is therefore important to further study risk factors and causes of premature birth in Suriname and explore the opportunities for implementation of both antenatal interventions (including improving lifestyle, early ultrasound, progesterone therapy, GBS screening, and cervical length measurements among high-risk women) and postnatal interventions (including early initiation of breastfeeding, kangaroo mother care and case management of neonatal sepsis) (WHO, 2014; Simmons et al., 2010). In addition, timely detection of congenital anomalies leads to better preparedness and management for parents and could potentially lead to improved neonatal outcomes (Goley et al., 2020; Seyi-Olajide et al., 2023). This study revealed that two-thirds of neonates with congenital anomalies were diagnosed after birth. Therefore, antenatal ultrasound in Suriname should be improved, starting with the implementation of routine 20-week ultrasound scan by qualified and skilled staff (Baardman et al., 2014). However, even if diagnosed timely, some congenital malformations may still result in perinatal death within the Surinamese healthcare context.

Implementation and re-evaluation of locally tailored guidelines such as management of HDP, (prevention of) preterm birth and neonatal resuscitation can effectively improve the quality of perinatal care in Suriname. In healthcare systems such as Suriname’s, implementation challenges arise from rapid patient turnover and high workload for healthcare professionals. Enhancing the quality of care necessitates an increase in the number of trained staff (both obstetric and pediatric). Finally, ensuring continuous access to equipment and trained personnel for neonatal life support is essential to reduce neonatal deaths in Suriname. The efficacy of programs like Helping Babies Breathe, a simulation-based neonatal resuscitation initiative for low-resource settings, underscores the positive impact of adequate training on perinatal outcomes (Niermeyer et al., 2020). Suriname has yearly simulation-based training, however, our study results emphasize the need for ongoing practice, frequent retesting, and refresher trainings (Bang et al., 2016).

This study found a delay in seeking care in nearly half of the reviewed cases. The precise reasons for this substantial contribution remain unknown. Nevertheless, the country’s deteriorating financial situation, the COVID-19 pandemic, and concomitant gaps in the health insurance system have heightened financial insecurity. This has resulted in delays in seeking healthcare and compromised access to care (Kodan et al., 2021; Pan American Health Organization, 2021). To address this issue and initiate a reduction in maternal and perinatal deaths, Suriname must ensure universal access to care for all pregnant women, eliminating financial barriers (Kodan et al., 2021).

Implementing the MPDSR cycle in low- and middle-income settings is particularly challenging, yet these settings present an opportunity for significant impact (Willcox et al., 2023). Incorrect implementation (e.g. an unsafe learning environment, stigmatization and inadequate leadership), however, can lead to pitfalls such as blame and litigation, resulting in adverse outcomes (Willcox et al., 2023; Pattinson et al., 2009). Currently, Suriname lacks a national policy for reviewing perinatal deaths. Our perinatal audits faced challenges including high caseloads, time-intensive preparation, incomplete documentation (clinical signs, vital parameters, and diagnostic results), missing files (especially for deaths outside hospitals), and limited resources (including staff shortages and diagnostic capacity such as microbial culture and autopsy). However, with this study, we showed that conduction of audits is possible within the Surinamese context. To successfully implement perinatal audits, we recommend case selection adjusted for the high caseloads and limited staff and establishing perinatal audit committees in every facility with adequate leadership for local and national data. Key to effective and sustainable perinatal audits is strong leadership from one or more audit committee(s) with senior clinicians responsible for protocol implementation and adherence. Furthermore, a learning-friendly and blame-free environment, positive feedback, and local implementation of recommendations to further motivate engagement (Willcox et al., 2023; Pattinson et al., 2009).While challenging, the implementation and evaluation of responses represent crucial steps to fulfill the MPDSR cycle (Pattinson et al., 2009). Successful examples underscore the potential for positive change, serving as a motivation for further engagement and fostering a positive perinatal death surveillance and response cycle (Willcox et al., 2023; Maaløe et al., 2019; Persson et al., 2013).

This study has limitations. First, this study did not include women without perinatal death as a reference group, which hampered comparisons. Secondly, the study period of six months period may not capture seasonal fluctuations in perinatal mortality, affecting generalizability of the reported PMR. Despite thorough surveillance, this is a facility-based surveillance and perinatal deaths occurring outside a healthcare facility (e.g. after homebirth or discharge of hospital) and going unregistered by vital statistics might have been missed, potentially leading to underreporting of the PMR. The cause of death was based primarily on clinical records and audit consensus, which may result in misclassification, particularly without advanced diagnostic investigations. Moreover, the audits were conducted within healthcare facilities and with prior knowledge of the outcomes, which may have biased the assessment toward proximal clinical factors, potentially underestimating the role of systemic or structural determinants. Staff may underreport delays, errors, and resource constraints during facility-based audits. Finally, due to the high caseload, we were unable to review every case of perinatal death during the study period. The strengths of this study lie in its prospective nature and the nationwide inclusion of perinatal death. Furthermore, this was the first study to describe perinatal audits in the country.

Based on the findings of our study, we have formulated recommendations to decrease perinatal mortality in Suriname, as outlined in Textbox 1.

## Conclusions for Practice

This study represents the first introduction of the MPDSR cycle in Suriname. Despite the challenges, we demonstrated that conduction of audits is achievable. Implementation of audits requires adequate leadership, installment of audit committees, proper time, staff and case-selection. To reduce prenatal mortality in Suriname, we recommend enhancements in both antenatal care and neonatal care including advancements in ultrasound, improved triage between high- and low-risk women, reducing the impact of HDP, and upgrading neonatal respiratory support. Furthermore, policymakers should prioritize improving access to care for all Surinamese women (mitigate the delay in seeking care) and sustainable implementation of the full MPDSR cycle.

Textbox 1 Recommendations to reduce perinatal mortality in Suriname.

1. Enhance perinatal mortality surveillance and incorporate stillbirths into the registration process.

2. Implement a comprehensive national perinatal death reduction strategy, which includes the MPDSR cycle: this involves establishing perinatal death audit committees in all healthcare facilities and prioritizing the implementation and evaluation of recommendations.

3. Diminish unexplained stillbirths through the implementation of recommendation 8, the performance of placenta pathology, and improvements in communication and documentation.

4. Mitigate the impact of HDP on adverse perinatal outcomes by implementing recommendations 5 and 8, refining triage for high- and low-risk pregnancies and promoting prevention and early recognition and treatment of HDP during antenatal care.

5. Improve the quality of antenatal care through enhanced communication between healthcare facilities, implementation of locally tailored guidelines, introduction of routine 20 weeks ultrasound scan by skilled staff, timely diagnosis of FGR and an increase of trained personnel.

6. Further study risk factors and causes of spontaneous premature birth in Suriname and explore the opportunities for implementation of both antenatal and postnatal interventions for premature birth.

7. Enhance neonatal life support and neonatal admission by ensuring the availability of equipment and trained staff in every facility, coupled with regular staff training.

8. Ensure universal access to healthcare for all pregnant women, eliminating financial barriers.

## Supplementary Information

Below is the link to the electronic supplementary material.

Supplementary material 1 (DOCX 56.3 kb)

## Data Availability

The authors can share anonymous data upon resendable request.
