# Supplementary material for: Perinatal Deaths in Suriname: A Nationwide Cohort Study on Causes of Death and Lessons Learned from Facility-Based Audits
Source: Matern Child Health J. 2026 May 14;30(6):843–54. doi: 10.1007/s10995-026-04280-1 (PMC13275568; doi:10.1007/s10995-026-04280-1)
Supplement: Supplementary file 1 — Supplementary material 1 (DOCX 56.3 kb) [file 10995_2026_4280_MOESM1_ESM.docx]

# **Supplementary files**

# **Article Title:** Perinatal deaths in Suriname: a nationwide cohort study on causes of death and lessons learned from facility-based audits

**Journal:** Maternal and Child Health Journal

**Authors**: Zita D. Prüst^1,2🖂^, Kim J.C. Verschueren^1,3^, Safir Liesdek^4^, Fernando Rigters^5^, Gieta Bhika-Kori^2^, Kitty W.M. Bloemenkamp^1^, Thomas van den Akker^3^, Lachmi R. Kodan^1,2,5^

^1^ Department of Obstetrics, Division Women and Baby, Birth Centre Wilhelmina’s Children Hospital, University Medical Centre Utrecht, Utrecht University

^2^ Department of Obstetrics and Gynecology, Academic Hospital Paramaribo (AZP),

^3^ Department of Obstetrics and Gynecology, Leiden University Medical Center, Leiden, the Netherlands

^4^ Department of Neonatology, Academic Hospital Paramaribo (AZP), Paramaribo, Suriname

^5^ Department of Obstetrics and Gynecology, ‘s Lands Hospitaal Paramaribo Suriname

^5^ Anton de Kom University of Suriname, Paramaribo, Suriname

^🖂^ **Corresponding author**

Zita Doré Prüst, MD, PhD-student

Department of Obstetrics, UMC Utrecht

Postal address: Postbus 85090 UMC Utrecht, 3508 AB Utrecht, the Netherlands

[zitaprust@gmail.com](mailto:zitaprust@gmail.com)

Phone: +31626303585

**Supplementary file 1. ICD-PM classification**

|  |  |
| --- | --- |
| **Antepartum death** | A1 Congenital malformations, deformations and chromosomal abnormalities  A2 Infection  A3 Antepartum hypoxia  A4 Other specified antepartum disorder  A5 Disorders related to fetal growth  A6 Antepartum death of unspecified cause |
| **Intrapartum death** | I1 Congenital malformations, deformations and chromosomal abnormalities  I2 Birth trauma  I3 Acute intrapartum event  I4 Infection  I5 Other specified intrapartum disorder  I6 Disorders related to fetal growth  I7 Intrapartum death of unspecified cause |
| **Neonatal death** | N1 Congenital malformations, deformations and chromosomal abnormalities  N2 Disorders related to fetal growth  N3 Birth trauma  N4 complications of intrapartum events  N5 Convulsions and disorders of cerebral status  N6 Infection  N7 Respiratory and cardiovascular disorders  N8 Other neonatal conditions  N9 Low birthweight and prematurity  N10 Miscellaneous  N11 Neonatal death of unspecified cause |
| **Maternal condition**  M1 Complications of placenta, cord and membranes  M2 Maternal complications of pregnancy  M3 Other complications of labor and delivery  M4 Maternal medical and surgical conditions  M5 No maternal condition | |

**Supplementary file 2. The classification of causes of perinatal deaths in Suriname, according to the ICD-PM**

|  |  | First maternal medical condition | | | | | |  | | |  |
| --- | --- | --- | --- | --- | --- | --- | --- | --- | --- | --- | --- |
|  |  | M1  Complications of placenta, cord and membranes | M2  Maternal complications of pregnancy | M3  Other complications of labor and delivery | M4  Maternal medical and surgical conditions | M5  No maternal condition | | Causes  Total  (%) | | |  |
| Causes of antepartum deaths |  |  | | | | | **66** | | | |  |
| A 1: Congenital malformations, deformations and chromosomal abnormalities |  |  |  |  |  | 4 | | 4 (7.1) | | |  |
| A 2: Infection |  |  |  |  |  |  | | 0 (0) | | |  |
| A 3: Antepartum hypoxia |  | 2 |  |  | 8 |  | | 10 (17.9) | | |  |
| A 4: Other specified antepartum disorder |  | 11 |  |  |  |  | | 11 (19.6) | | |  |
| A 5: Disorders related to foetal growth |  | 5 |  |  | 3 | 0 | | 8 (14.3) | | |  |
| A 6: Foetal death of unspecified cause |  | 1 | 1 |  | 4 | 17 | | 23 (41.1) | | |  |
| Unclassified antepartum deaths |  |  |  |  |  |  | | 10 | | |  |
| Causes of intrapartum deaths |  |  | | | | | | | **13** | |  |
| I 1: Congenital malformations, deformations and chromosomal abnormalities |  |  |  |  |  | 1 | | 1 (7.7) | | |  |
| I 2: Birth trauma |  |  |  |  |  |  | | 0 (0.0) | | |  |
| I 3: Acute intrapartum event |  | 2 |  |  |  | 1 | | 3 (23.1) | | |  |
| I 4: Infection |  | 1 |  |  |  |  | | 1 (7.7) | | |  |
| I 5: Other specified intrapartum disorder |  | 4 |  | 2 |  |  | | 6 (42.6) | | |  |
| I 6: Disorders related to foetal growth |  |  | 1 |  |  |  | | 1 (7.7) | | |  |
| I 7: Intrapartum death of unspecified cause |  |  |  |  |  | 1 | | 1 (7.7) | | |  |
| Unknown timing of death | |  |  |  |  |  | | **4** | | |  |
| U 1: Congenital malformations, deformations and chromosomal abnormalities | |  |  |  |  |  | |  | | |  |
| U 2: Birth trauma | |  |  |  |  |  | |  | | |  |
| U 3: Acute antepartum/intrapartum event | |  |  |  |  |  | |  | | |  |
| U 4: Infection | |  |  |  |  |  | |  | | |  |
| U 5: Other specified intrapartum disorder | |  |  |  |  |  | |  | | |  |
| U 6: Disorders related to foetal growth | |  |  |  |  |  | |  | | |  |
| U 7: Stillbirth of unspecified cause | |  |  |  |  | 2 | | 2 | | |  |
| Unclassified stillbirths of unknown timing | |  |  |  |  |  | | 2 | | |  |
| Causes of neonatal deaths | | | | | | | | | **49** |  |  |
| N 1: Congenital malformations, deformations and chromosomal abnormalities | |  | 2 |  | 1 | 11 | | 14 (28.6) | | |  |
| N 2: Disorders related to fetal growth | |  |  |  |  |  | |  | | |  |
| N 3: Birth trauma | |  |  |  |  |  | |  | | |  |
| N 4: Complications of intrapartum events | |  | 1 |  | 1 | 3 | | 5 (10.2) | | |  |
| N 5: Convulsions and disorders of cerebral status | |  |  |  |  |  | |  | | |  |
| N 6: Infection | |  | 7 |  | 3 | 1 | | 11 (22.4) | | |  |
| N 7: Respiratory and cardiovascular disorders | |  | 6 |  | 4 |  | | 10 (20.4) | | |  |
| N 8: Other neonatal conditions | |  |  |  | 1 | 1 | | 2 (4.1) | | |  |
| N 9: Low birthweight and prematurity | |  | 6 |  | 1 |  | | 7 (14.3) | | |  |
| N 10: Miscellaneous | |  |  |  |  |  | |  | | |  |
| N 11: Neonatal death of unspecified cause | |  |  |  |  |  | |  | | |  |
| Unclassified neonatal deaths | |  |  |  |  |  | |  | | |  |
| Maternal condition total  (%) | | **26 (21.7)** | **24 (20.0)** | **2 (1.7)** | **26 (21.7)** | **42 (35.0)** | | **132** | | |  |
| Legend  Most common causes and their classification:  Antepartum:   - Foetal Growth restriction due to placental insufficiency: A5M1 - Foetal Growth restriction among women with Hypertensive Disorders of Pregnancy (HDP): A3M1 - Placental abruption: A4M1 (if in combination with HDP, then with M4 as second maternal condition) - Unspecified antepartum stillbirth: A6M5   Intrapartum:   - Placental abruption: I5M1 (if in combination with HDP, then with M4 as second maternal condition) - Uterine rupture: I5M3 - Spontaneous extreme prematurity: I6M1   Neonatal:   - Intrapartum asphyxia: N4 (M4 if in combination with HDP; M5 if without a maternal condition) - Pulmonary haemorrhage in neonatal sepsis: N6 - Necrotizing enterocolitis: N8 - Extreme prematurity: N9 | | | | | | | | | | | |

**Supplementary file 3. Perinatal audit form: modifiable care factors and recommendations**

**General information**

Date:

Attendees:

Moderator:

Notes by:

Location:

Case number:

1. **What happened?**
2. **What happened?**

*Case summery:*

1. **What were the circumstances in which the perinatal death occurred?**

*Where:*

*Involved healthcare workers:*

*Other circumstances:*

1. **What went right and what could have done better in future?**

**3a. What went right and on what level did that happen?**

|  |  |
| --- | --- |
| *Patient related* |  |
| *Team/Communication related* |  |
| *Care giver/Task related* |  |
| *System/Management related* |  |

**3b. What were potential modifiable factors and on what level did they occur?**

|  |  |
| --- | --- |
| *Patient related* |  |
| *Team/Communication related* |  |
| *Quality of care related* |  |
| *System/Management related* |  |

1. **What is the relation between the modifiable care factor and death?**

|  | None |  | Probable |
| --- | --- | --- | --- |
|  | Unlikely |  | Very probable |
|  | Possible |  | Unable to classify |

Explanation:

1. **What are the conclusions/lessons learned?**


1. **What can be done to prevent the modifiable care factor from occurring again?**
